# Nondestructive Identification of Rare Trophoblastic Cells by Endoplasmic Reticulum Staining for Noninvasive Prenatal Testing of Monogenic Diseases

**DOI:** 10.1002/advs.201903354

**Published:** 2020-02-13

**Authors:** Yifang Huang, Bo Situ, Liping Huang, Yingsi Cao, Hong Sui, Xinyi Ye, Xiujuan Jiang, Aifen Liang, Maliang Tao, Shihua Luo, Ye Zhang, Mei Zhong, Lei Zheng

**Affiliations:** ^1^ Department of Laboratory Medicine Nanfang Hospital Southern Medical University Guangzhou 510515 P. R. China; ^2^ Guangdong Engineering and Technology Research Center for Rapid Diagnostic Biosensors Nanfang Hospital Southern Medical University Guangzhou 510515 P. R. China; ^3^ Guangdong Provincial Key Laboratory of Single Cell Technology Application Guangzhou 510515 P. R. China; ^4^ Department of Obstetrics and Gynecology Nanfang Hospital Southern Medical University Guangzhou 510515 P. R. China; ^5^ Department of Laboratory Medicine Dongguan Kanghua Hospital Dongguan 523080 P. R. China

**Keywords:** monogenic diseases, nondestructive identification, noninvasive prenatal testing, single‐cell isolation, trophoblastic cells

## Abstract

Noninvasive prenatal detection of monogenic diseases based on cell‐free DNA is hampered by challenges in obtaining a sufficient fraction and adequate quality of fetal DNA. Analyzing rare trophoblastic cells from Papanicolaou smears carrying the entire fetal genome provides an alternative method for noninvasive detection of monogenic diseases. However, intracellular labeling for identification of target cells can affect the quality of DNA in varying degrees. Here, a new approach is developed for nondestructive identification of rare fetal cells from abundant maternal cells based on endoplasmic reticulum staining and linear discriminant analysis (ER‐LDA). Compared with traditional methods, ER‐LDA has little effect on cell quality, allowing trophoblastic cells to be analyzed on the single‐cell level. Using ER‐LDA, high‐purity of trophoblastic cells can be identified and isolated at single cell resolution from 60 pregnancies between 4 and 38 weeks of gestation. Pathogenic variants, including –^SEA^/ deletion mutation and point mutations, in 11 fetuses at risk for α‐ or β‐thalassemia can be accurately detected by this test. The detection platform can also be extended to analyze the mutational profiles of other monogenic diseases. This simple, low‐cost, and noninvasive test can provide valuable fetal cells for fetal genotyping and holds promise for prenatal detection of monogenic diseases.

## Graphical abstract

An endoplasmic reticulum staining and linear discriminant analysis single‐cell test for detecting rare trophoblastic cells in Pap samples from pregnancies whose fetuses are at risk for monogenic disorders is presented. The antibody‐free, simple, low‐cost assay enables genomic analysis of fetal trophoblastic cells with high performance, which can serve as a promising tool for prenatal detection of monogenic diseases.

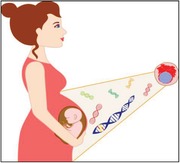

## 1 Introduction

Investigation of fetal genetic information in early pregnancy is of great importance for the prevention of genetic diseases. Invasive procedures, such as amniocentesis (AC, 16 weeks of gestational age) and chorionic villous sampling (CVS, 10–12 weeks of gestational age), are the current gold standards for diagnosing fetal genetic diseases.^[^[qv: 1,2]^]^ However, these invasive procedures may increase the risk of fetal infection and miscarriage.^[^[qv: 2]^]^ Therefore, great efforts have been made to develop noninvasive approaches for prenatal genetic testing.^[^[qv: 3]^]^ Cell‐free fetal DNA (cffDNA) based testing has high sensitivity and specificity in detecting common fetal chromosomal aneuploidies and has widely being implemented in clinical practice.^[^[qv: 4]^]^ However, cffDNA based assays for the detection of monogenic diseases, a category of diseases caused by point mutations or micro‐deletions in pathogenic genes, are limited by their complicated steps of next‐generation sequencing (NGS) and parental haplotyping.^[^[qv: 5]^]^ Furthermore, the highly fragmented nature (≈200 bp) and low fetal fraction (with medians of 10–15%) of cell free DNA in the maternal circulation may lead to unreliable results in fetal genotyping.^[^[qv: 6,7]^]^ Intact fetal cells for prenatal testing have attracted a great deal of attention.^[^[qv: 8,9]^]^ Pure fetal genomic DNA can be obtained from individual fetal cells, allowing the determination of fetal genotype without statistical assessment. Furthermore, fetal cells with the entire fetal genome can provide fetal DNA with high integrity, making them suitable for use in the analysis of single nucleotide variations. In recent decades, many studies have focused on isolating circulating fetal cells from maternal peripheral blood.^[^[qv: 10–12]^]^ However, these efforts have had limited success because it is challenging to find extremely rare fetal cells among abundant maternal blood cells (≈6 fetal cells and 10^9^ maternal cells per mL of blood).^[^[qv: 13]^]^ In contrast to circulating fetal cells, fetal trophoblastic cells in maternal reproductive tract have been demonstrated to be a promising cell source for noninvasive genetic investigation.^[^[qv: 14,15]^]^ These fetal cells are released from the conceptus by an unknown mechanism and their number is much higher than that of circulating fetal cells (hundreds to thousands of fetal cells per specimen and about one fetal cell among 2000 maternal cells) (Figures S1 and S2, Supporting Information).^[^[qv: 16,17]^]^ However, although much progress has been made in understanding their cellular phenotype,^[^[qv: 14]^]^ there is still a lack of investigation on the molecular profiling of these cells. As trophoblastic cells are mixed with a large number of maternal epithelial cells, purification and identification processes are indispensable.^[^[qv: 14,15]^]^ Advances in isolation technology such as magnetic activated cell sorting based assays have allowed the separation of trophoblastic cells with little cell damage.^[^[qv: 14,15]^]^ However, traditional methods for the identification of such cells often require cumbersome steps of cell fixation and penetration. The intracellular labeling process may potentially affect the quality of DNA, which can influence the success of downstream molecular analysis.^[^[qv: 18,19]^]^ Detection of the cell‐surface markers, such as HLA‐G, seems to be a solution to address this problem. However, HLA‐G is not specific enough and its expression is reduced in preeclamptic pregnancies.^[^[qv: 20,21]^]^ This may lead to low fetal cell purity.

**Figure 1:**
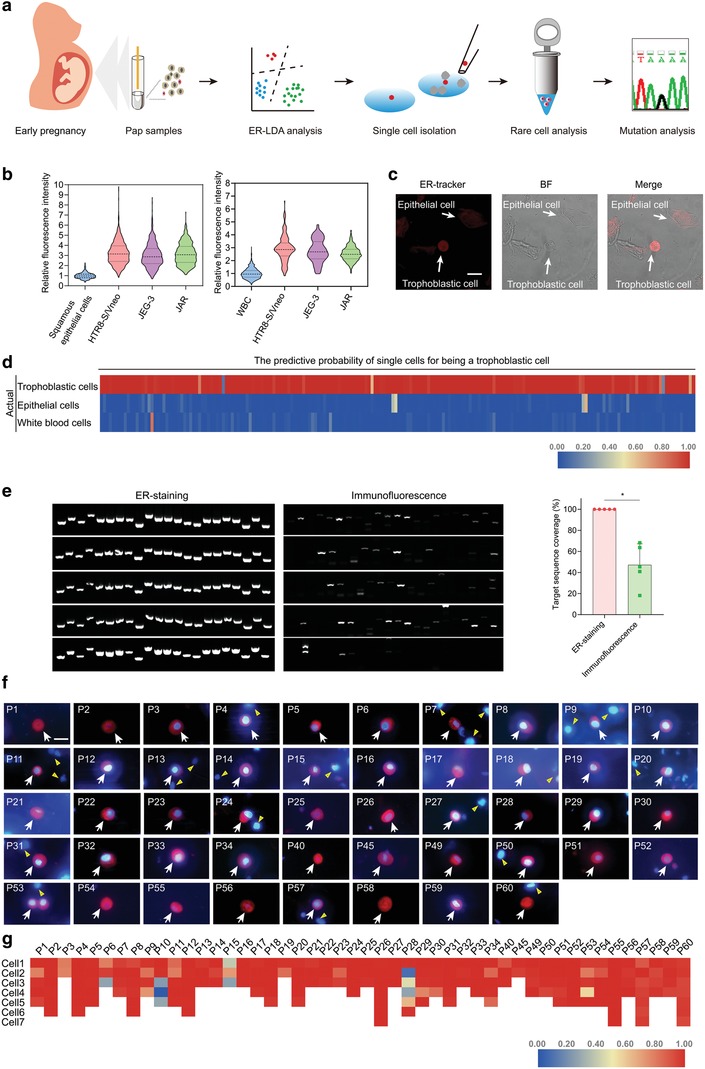
Development of ER‐LDA for analyzing rare trophoblastic cells. a) The working principle of ER‐LDA cell‐based test. ER^high^/DAPI^+^ cells are analyzed by LDA, validated by STR, and isolated at single‐cell level for sequencing. ER^high^ cells, candidate trophoblastic cells with deep staining of endoplasmic reticulum. DAPI, 4′,6‐diamidino‐2‐phenylindole. b) The relative fluorescence intensity of ER‐tracker in three trophoblastic cell lines, HTR8‐S/Vneo, JEG‐3, and JAR, and in squamous epithelial cells and white blood cells taken from healthy female donors. CFDA‐SE pre‐labeled trophoblastic cells are mixed with a proportion of squamous epithelial cells or white blood cells, stained with ER‐tracker and imaged by a fluorescence microscope. The fluorescence intensity of each single cell is quantified by Image J software, and the relative fluorescence intensity between trophoblastic cells and squamous epithelial cells or white blood cells is calculated. Lines represent the mean values and the interquartile range of 5 replicates. c) ER^high^ trophoblastic cells imaged by confocal microscopy. CFDA‐SE pre‐labeled trophoblastic cells can be easily identified by ER‐Tracker in spiked‐in samples. Scale bar, 20 µm. d) The LDA predictive probability of 200 trophoblastic cells, 200 squamous epithelial cells and 200 leukocytes in test cohort. e) The coverage of WGA products assessed by the amplification of 22 genomic loci. Five single cells identified by ER‐LDA or β‐HCG intracellular labeling are used for each test and five replicates are performed (Mean ± SD). Mann‐Whitney U test, *P < 0.05. f) Representative images of ER^high^ trophoblastic cells in 48 Pap samples. Cells are stained with ER‐Tracker and DAPI. ER‐Tracker is shown in red; DAPI is shown in blue. The white arrows indicate the target trophoblastic cells and the yellow triangles show the maternal cells. Before single‐cell isolation, these cells are mixed with a proportion of maternal cells. Scale bar, 20 µm. g) The LDA predictive probability of 209 candidate trophoblastic cells from 48 Pap samples. Two to 7 single cells per sample are analyzed and collected for WGA.

In this study, we demonstrate a novel cell‐based noninvasive prenatal testing system, in which rare trophoblastic cells in Papanicolaou smears (Pap) can be nondestructively identified and isolated at single‐cell level, thus providing pure fetal DNA with high integrity for fetal genotyping (**Figure** **1**a). We showed that trophoblastic cells produced a higher fluorescence signal compared to maternal cells in endoplasmic reticulum (ER) staining. Trophoblastic cells could be clearly distinguished from abundant maternal cells by ER based‐LDA analysis (ER‐LDA). Compared with intracellular labeling in vivo, ER‐LDA has minimal effect on cell quality, thus ensuring the successful genotyping at single‐cell level. Importantly, ER‐LDA cell‐based test showed high performance on the prenatal detection of thalassemia, one of the most common recessive monogenic diseases in southern China.^[^[qv: 22]^]^ This cell‐based test may also be extended to the detection of other monogenic disorders.

## 2 Results

### 2.1 Establishment of ER‐LDA Assay for Identifying Rare Trophoblastic Cells

To nondestructive identification of rare trophoblastic cells in Pap samples, a novel assay based on endoplasmic reticulum staining and linear discriminant analysis (ER‐LDA) was developed. The continuous secretion of human chorionic gonadotropin (hCG) and a high level of progesterone are the hallmarks of trophoblastic cells.^[^[qv: 23,24]^]^ An important characteristic of these secretory cells is that their rough ER is particularly abundant, presenting an opportunity to distinguish fetal trophoblastic cells from maternal cells without immunolabelling. To validate this assumption, trophoblastic cells from trophoblastic cell lines, with squamous epithelial cells and white blood cells (WBCs), two major cell types in Pap specimens, were treated with ER‐Tracker, a highly selective dye for ER staining. We found that the fluorescence intensity of ER‐Tracker in trophoblastic cells was about 3.13 ± 1.13 (mean ± s.d.) and 2.64 ± 0.73 (mean ± s.d.) fold higher than that in squamous epithelial cells and WBCs, respectively (Figure 1b). In spiked‐in samples (containing 500 trophoblastic cells, ≈200 000 squamous epithelial cells and WBCs), trophoblastic cells with a remarkable ER fluorescence could be easily identified by confocal imaging (Figure 1c).

Subsequently, we then used LDA analysis to improve the discrimination between trophoblastic and nontrophoblastic cells. The ER fluorescence and cell size of individual cells were used for LDA analysis. In the training cohort (trophoblastic cells = 1148, squamous epithelial cells = 258, WBCs = 948), the LDA showed a sensitivity of 96.51%, a specificity of 99.45% and an accuracy of 98.00% for discrimination of trophoblastic cells versus nontrophoblastic cells. Subsequently, a test cohort (trophoblastic cells = 200, squamous epithelial cells = 200, WBCs = 200) was used to demonstrate the capability of ER‐LDA to identify trophoblastic cells among nontrophoblastic cells. An accuracy of 98.83% was obtained for identification of trophoblastic cells. Of the 198 cells predicted to be trophoblastic cells, the predictive probability of 193 (97.47%) cells was greater than 0.9 (Figure 1d). With the aid of ER‐LDA, trophoblastic cells with a bright fluorescence could be isolated at single‐cell level from spiked‐in samples (Figure S3a, Supporting Information). These results indicate that the ER‐LDA assay, with its high accuracy, is suitable for the rapid identification of fetal trophoblastic cells in Pap samples.

To test whether ER‐LDA affects the quality of rare trophoblastic cells for molecular analysis, single JEG‐3 cells identified by ER‐LDA in spiked‐in sample were isolated and used for whole genome amplification (WGA). WGA products were used as template to amplify 22 genetic loci across 22 chromosomes to assess the genomic coverage. We found that all the 22 genetic loci in five single cells showed successful amplifications, which supports their high genomic coverage (Figure 1e). In contrast, only 4–12 loci were detectable in single cells identified by immunofluorescence, indicating the low DNA quality (Figure 1e).

### 2.2 Isolation and Characterization of Rare Fetal Cells from Clinical Samples

We subsequently examined the feasibility of using ER‐LDA in the investigation of clinical samples. Using this assay, a range of two to 19 putative fetal cells could be identified in 60 samples with gestational ages ranging from 4 to 38 weeks (Table S1, Supporting Information). As expected, putative trophoblastic cells showed a much brighter ER fluorescence (2‐ to 5‐fold) than that of background cells (Figure 1f). All of these cells showed trophoblast‐like morphology: round or oval shape in size of 16.6 ± 1.96 µm and with high nucleus‐to‐cytoplasm. The trophoblast‐like cells with high ER fluorescence and medium size were clearly discriminated and their probability of being trophoblastic cells was obtained by ER‐LDA (Figure 1g). Among 209 ER^high^ single cells in 48 samples, 197 (94.26%) cells showed a probability greater than 0.7 and 180 (86.12%) cells showed a probability greater than 0.9 of being trophoblastic cells (Figure 1g), suggesting their possible fetal origin. Additionally, the number of these single cells was not affected by the gestational week and maternal age, indicating that the isolation of fetal cells is applicable to most of pregnant women (Figure S4, Supporting Information). These observations suggest that the ER‐LDA assay is capable of identifying trophoblast‐like cells from clinical samples.

**Figure 2:**
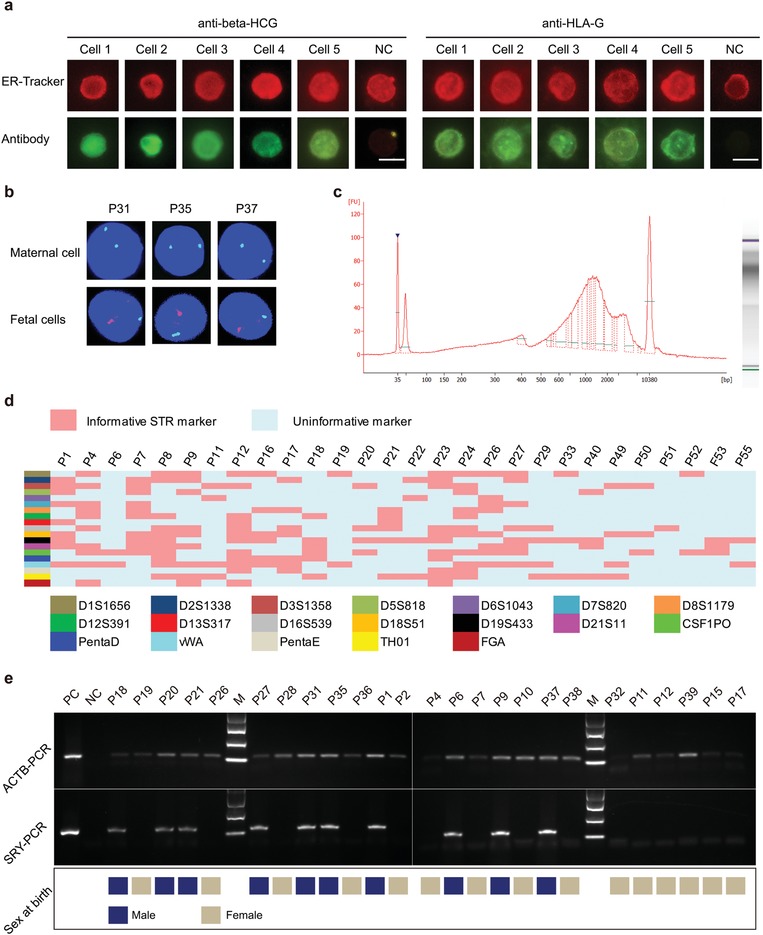
Confirmation of the fetal origin of ER^high^ trophoblastic cells. a) Representative immunostaining results of candidate cells isolated from sample P26. Twelve single trophoblastic cells are isolated, six cells are stained with β‐HCG and six stained with HLA‐G. NC, negative control, cells are not stained with antibodies. Scale bar, 15 µm. b) ER^high^ trophoblastic cells isolated from seven pregnancies with a male fetus processed with FISH analysis. Representative results of single cells from P31, P35, and P37 are shown. Y chromosome signal is observed in these cells. Green fluorescence and red fluorescence, respectively, represent chromosome X and chromosome Y. c) The fragment size of the WGA product of P24 assessed by Agilent 2100 bioanalyzer. d) The STR profiles of maternal genomic DNA and fetal DNA amplified from rare fetal cells. 28 WGA samples with concentrations greater than 300 ng µL^−1^ are used for STR analysis. The red squares represent the informative loci between fetal and maternal cells. e) SRY‐PCR for detecting chromosome Y. SRY amplicons are detected in pregnancies with a male fetus. The actin beta (ACTB) gene is used as the internal reference.

We next sought to characterize the expression of β‐hCG and HLA‐G, trophoblastic cell markers, on ER^high^ single cells. These cells showed a high ER fluorescence signal and all cells were predicted to be trophoblastic cells with LDA predictive probabilities ranging from 0.65 to 1.00. We observed that most of these cells were β‐hCG and HLA‐G positive, indicative of the high accuracy of ER‐LDA in trophoblastic cells identification (**Figure** **2**a and Figure S5, Supporting Information). Furthermore, seven samples from pregnancies with a male fetus were processed with FISH analysis. We found that ER^high^ cells separated from these samples showed positive probe binding for the Y chromosome, suggesting their fetal origin (Figure 2b).

In addition, single ER^high^ cells were also isolated and subjected to whole genome amplification. The quality and integrity of WGA products was assessed using an Agilent 2100 bioanalyzer. The mean concentration of these WGA samples was 502.95 ± 269.25 ng µL^−1^ and the fragment size ranged from 500 to 5000 bp (Figure 2c). These values for concentration and integrity were considered to be higher than those of cell‐free DNA extracted from maternal blood.^[^[qv: 7]^]^ The successful amplification of 22 loci on different chromosomes also indicated a high genomic coverage of WGA (Figure S3b, Supporting Information). These results demonstrated that the DNA quality of WGA samples amplified from rare ER^high^ cells was adequate for downstream genetic analysis.

Using WGA products as template, short tandem repeat (STR) profiling, a gold standard for cell identification, was also performed to further confirm the fetal origin of ER^high^ cells. As shown in Table S2 (Supporting Information), 90.6% samples were found to be harboring two to eleven informative STR markers that distinguish the fetus from the mother (Figure 2d). In particular, loci with three or more alleles were not detected in these samples, suggesting that the target cells we collected were pure fetal cells. To verify the consistency of LDA results and STR results, samples containing cells with a low LDA predictive probability value (three of six cells in P28, three of five cells in P10, and two of three cells in P15 were lower than 0.5) were also tested by STR. Consistent with the LDA results, the STR genotype of these samples showed the existence of maternal cell contamination (Figure S6, Supporting Information). These data indicated that ER‐LDA assay has a high accuracy in the identification of fetal trophoblastic cells in Pap samples and the probability of 0.65 can be used as a threshold to distinguish trophoblastic cells from maternal cells. SRY‐PCR was also conducted to detect the presence of the Y chromosome. We found that SRY amplicons could be detected in pregnancies with a male fetus (Figure 2e). The gender of the fetuses was consistent with the subsequent gender at birth. Altogether, the above results further indicated that the ER^high^ single cells we isolated were indeed fetal cells. As a comparison, we used STR to assess the purity of cells sorted by antibody‐based flow cell sorting, a traditional method. We found that three of four samples (75%) showed the presence of maternal cell contamination (Figure S6, Supporting Information).

### 2.3 Application in Thalassemia Detection

**Figure 3:**
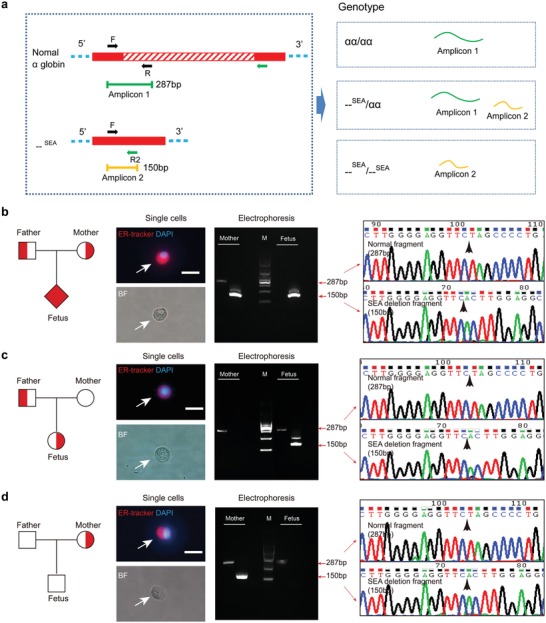
Sequencing of candidate fetal cells from three pregnancies whose fetuses are at risk for –^SEA^/ thalassemia. a) Short fragment Gap‐PCR for detecting –^SEA^/ mutation. A fragment of 150 bp is amplified from the chromosome with the –^SEA^/ deletion and a fragment of 287 bp is amplified from the normal chromosome. b) For patient P30, the mother is heterozygous for –^SEA^/ deletion, while the fetus is a homozygote. c) For patient P4, –^SEA^/ heterozygous mutation is found in rare fetal cells but not found in maternal DNA. d) For patient P18, the mother is heterozygous for –^SEA^/ deletion, while the fetus is normal. BF, bright field. Scale bar, 20 µm. The white arrow indicates the single cells isolated from Pap samples and the black arrow indicates the breakpoint of the mutation. Fully symbols indicate affected members who carry homozygous mutation. Half‐filled symbols indicate members who carry heterozygous mutation. Squares indicate males, circles indicate females, and diamonds indicate individuals of unknown gender.

Thalassemia is one of the most common monogenic diseases, with a high prevalence in southern China.^[^[qv: 22]^]^ To investigate whether the rare fetal trophoblastic cells can be applied to noninvasive prenatal testing for thalassemia, 11 pregnancies whose fetuses were at risk for thalassemia were recruited (**Table** **1**). In these cases, pregnant women or their spouses were found to carry pathogenic variants for thalassemia. The average gestational age of these pregnancies was 18.9 weeks (ranging 4–32 weeks). We first evaluated the feasibility of rare fetal trophoblastic cells for the detection of –^SEA^/ thalassemia, a common form of α‐thalassemia. The high‐integrity of fetal DNA obtained from intact trophoblastic cells allows the detection of –^SEA^/ thalassemia with only two simple steps: short fragment Gap‐PCR and Sanger sequencing. Using short fragment Gap‐PCR, a fragment of 150 bp was amplified from the chromosome with the –^SEA^/ deletion and a fragment of 287 bp was amplified from the normal chromosome.^[^[qv: 25]^]^ Therefore, homozygotes for the –^SEA^/ deletion showed only a 150 bp fragment, carriers have both fragments and normal individuals have only the 287 bp fragment (**Figure** **3**a). Trophoblastic cells from five cases whose fetuses were at risk for –^SEA^/ deletion were analyzed. For example, five and four trophoblastic cells were retrieved from subjects P5 and P30, respectively. For these subjects, a homozygous mutation, –^SEA^/–^SEA^ in α‐gene cluster associated with hemoglobin Bart's hydrops fetalis syndrome was detected in the fetuses. Gap‐PCR showed only the –^SEA^/ deletion fragment and the deletion breakpoint of the –^SEA^/ mutation could be clearly identified in Sanger sequencing (Figure 3b and Figure S7a, Supporting Information). These results were consistent with those of AC/CVS. In P4, six trophoblastic cells were identified and a paternal heterozygous variant, –^SEA^/*αα*, was identified in trophoblastic cells (Figure 3c and Table 1). The normal genotype was found in the mother, suggesting that the detection of ER^high^ trophoblastic cells is capable of identifying paternal variants in the fetus. It should be noted that the gestational age of P4 was four weeks, indicating the feasibility of our cell‐based test for early prenatal testing. In P18, rare trophoblastic cells exhibited the same genotype as the father, *αα*/*αα*, however, the mother was a –^SEA^/ heterozygote (Figure 3d and Table 1). Consistent with these results, the phenotype of the fetus presented as normal after birth. We also found that the genotype of rare trophoblastic cells in one case, P1, shared the same genotype as its mother (Figure S7b, Supporting Information). STR analysis was useful in affirmation of the fetal origin of these cells. Furthermore, the mutational status of P1 was found to be consistent with that of AC/CVS. Results of all cases were confirmed by invasive or postnatal tests and the genotype of fetuses, including abnormal fetuses and normal fetuses, was accurately detected by our cell‐based test. These results provide evidence that this trophoblastic cell‐based platform can serve as a reliable tool for –^SEA^/ thalassemia detection.

**Table 1:** Results of thalassemia related mutations in candidate fetal trophoblastic cells

Patient	Age	Gestational age (week)	Single cells	Genotypes for	Father	Mother	Fetus (rare‐cell analysis)	AC/CVS/ postnatal testing
P1	29	22^+1^	5	α‐globin	–^SEA^/ *α α*	–^SEA^/ *α α*	–^SEA^/ *α α*	–^SEA^/ *α α*
P2	25	23^+6^	6	β‐globin	normal	CDs41/42(‐TCTT)	normal	normal
P4	31	4^+4^	6	α‐globin	–^SEA^/ *α α*	*α α*/ *α α*	–^SEA^/ *α α*	–^SEA^/ *α α*
P5	30	29^+2^	5	α‐globin	–^SEA^/ *α α*	–^SEA^/ *α α*	–^SEA^/ –^SEA^	–^SEA^/ –^SEA^
P18	19	13^+6^	5	α‐globin	α α/ *α α*	–^SEA^/ *α α*	*α α*/ *α α*	normal
P30	25	32^+6^	4	α‐globin	–^SEA^/ *α α*	–^SEA^/ *α α*	–^SEA^/ –^SEA^	–^SEA^/ –^SEA^
P50	17	10	4	β‐globin	normal	CDs41/42(‐TCTT)	CDs41/42(‐TCTT)	CDs41/42(‐TCTT)
P54	27	17^+5^	5	β‐globin	IVS‐II‐654(C > T)	IVS‐II‐654(C > T)	normal	normal
P58	27	17^+2^	4	β‐globin	IVS‐II‐654(C > T)	−28(A > G)	−28(A > G)	−28(A > G)
P59	29	32	4	β‐globin	normal	IVS‐II‐654(C > T)	normal	normal
P60	27	16	7	α‐and β‐globin	–^SEA^/ *α α*, −28(A > G)	normal	−28(A > G)	−28(A > G)

**Figure 4:**
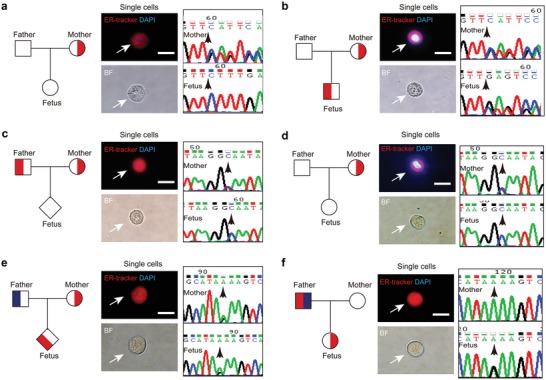
Sequencing of candidate fetal cells from six pregnancies whose fetuses are at risk for β‐thalassemia. a) The sequencing results suggested that the fetus is normal for patient P2, while the mother harbors a CDs41‐42(‐TCTT) mutation. b) The fetus and mother harbor a CDs41‐42(‐TCTT) mutation for patient P50. c) For patient P54, the father and the mother are heterozygous for IVS‐II‐654(C > T), while the fetus is normal. d) For patient P59, The mother is heterozygous for IVS‐II‐654(C > T), while the fetus is normal. e) For patient P58, the mother and the fetus are heterozygous for −28(A > G). Another variant, IVS‐II‐654(C > T), is present in the father. f) For patient P60, the mother is normal, while the fetus is heterozygous for −28(A > G). The father harbors two variants, –^SEA^/*αα* in α‐gene cluster and −28(A > G) in HBB gene. Scale bar, 20 µm. The white arrow indicates the single cells isolated from Pap samples and the black arrow indicates the location of the mutations. Fully symbols indicate affected members who carry homozygous mutation. Half‐filled symbols indicate members who carry heterozygous mutation. Red symbols indicate individuals with the HBB mutations, and blue symbols indicate individuals with another mutation in the same family. Squares indicate males, circles indicate females, and diamonds indicate individuals of unknown gender. Single cells isolated from Pap samples are shown.

Several cases with β‐thalassemia were also recruited and analyzed. Four to seven trophoblastic cells were identified, with the LDA predictive probability value greater than 0.85 in these cases. In P2, the fetus had inherited a normal allele from the father, while the mother harbors a CDs41‐42(‐TCTT) variant which can lead to a mild form of β‐thalassemia (**Figure** **4**a, Table 1). In P50, CDs41‐42(‐TCTT) was detected in the fetus. This deletion variant was also present in the mother, who had been diagnosed with β^0^‐thalassemia (Figure 4b and Table 1). The genotype of the fetuses detected by trophoblastic cell‐test in P2 and P50 was consistent with those of postnatal tests. In P54, we found normal allele in the fetus, which was consistent with the AC/CVS result, while the parents carry the mutation of IVS‐II‐654(C>T) in the HBB gene associated with β^+^‐thalassemia (Figure 4c and Table 1). In subject P59, paternal normal allele was identified in trophoblastic cells, while the mother had a molecular diagnosis of IVS‐II‐654(C > T) heterozygote (Figure 4d and Table 1). In P58, a pathogenic variant, −28(A > G) in the HBB gene associated with β^+^‐thalassemia was detected in the fetus. This variant was present in the mother and another variant, IVS‐II‐654(C > T), was present in the father (Figure 4e and Table 1). Notably, in P60, the fetus had inherited a normal allele from the mother and a pathogenic variant, −28(A > G), from the father (Figure 4f and Table 1). The father was found to harbor two variants, –^SEA^/*αα* in α‐ gene cluster and −28(A > G) in HBB gene. The results of P59, P58, and P60 were also consistent with those of AC/CVS. These results indicated that the cell‐based test is also suited for the prenatal detection of β‐thalassemia or β‐thalassemia combined with –^SEA^/‐thalassemia. No false‐negative and false‐positive results were detected in these cases. Altogether, regardless of the parents' genotype, this simple cell‐based test could accurately detect thalassemia‐associated variants in fetuses, including deletion mutations and point mutations.

### 2.4 Whole Exome Sequencing for the Detection of Other Monogenic Diseases

The high performance of trophoblastic cell‐based test in thalassemia detection prompted us to test its feasibility in the detection of other monogenic diseases. We performed whole exome sequencing (WES) on rare fetal cells from five samples (Figure S8a, Supporting Information). Intact cells provided high‐quantity amplified DNA for the successful library construction and sequencing. A mean of 143267 SNVs and 22407 InDels could be detected in these samples (Figure S8c, Supporting Information). A quality peak of 60 was observed for most variants in the genotype quality analysis and the average percentage of low quality variants (<60) was about 19.93%.^[^[qv: 10]^]^ Importantly, the data on variants covered an average total number (HGMD: 763, Clinvar: 4047 and OMIM: 3307) of disease‐associated genes across the whole genome, which allowed us to investigate the pathogenic mutations for diagnosing common monogenic diseases. For the 52 most common monogenic disease‐associated genes, 77 variants in 39 genes were identified in these samples and plotted (Figure S8d, Supporting Information). These results demonstrated that the rare fetal cells showed good performance in detection of SNVs and the variants were considered of high quality. In addition, rare fetal cells could be successfully applied to analysis of CNVs (copy number variations), indicating their potential role in the detection of chromosome‐related abnormalities (Figure S8b, Supporting Information). Overall, rare fetal cells may also be suitable for use in identifying inheritable mutations in a variety of monogenic diseases by high throughput sequencing.

## 3 Discussion

The technical advances in this work include nondestructive identification of rare trophoblastic cells for providing pure and high‐quality of fetal DNA; characterization of the phenotype and confirmation of fetal origin of rare trophoblastic cells for accurate genotyping; a feasibility analysis of using the cell‐based test for the detection of potential pathogenic mutations in the targeted genes of thalassemia. The presented antibody‐free, simple, low‐cost assay can be used to expand the current test for the detection of many more monogenic diseases, including recessive diseases and dominant diseases.

The collection of individual cells with high quality is critical for successful molecular analysis. However, the cumbersome and complicated processing required for intracellular labeling may result in a decline in the quality of target cells. Rapid screening of rare cells by exploiting the differences in biological activity of cellular organelles between target and background cells can be regarded as a sensible choice.^[^[qv: 26]^]^ Trophoblasts with high ER activity represent an active secretory subset of trophoblastic cells in Pap samples from pregnant women.^[^[qv: 27]^]^ This distinctive feature enables the discrimination of rare fetal cells from maternal cells by simple steps of ER staining and LDA analysis. The enrichment‐free and one‐step labeling assay without cell fixation and penetration is considerably reduce damage to rare fetal cells. Importantly, using ER‐LDA, we have achieved a high accuracy in distinguishing fetal trophoblastic cells from maternal cells. In real Pap samples, two to 19 candidate trophoblastic cells could be rapidly identified and the single cells we isolated from about 90.6% samples were validated as pure fetal cells. Furthermore, consistent with previous reports, trophoblastic cells could be found at an early gestational age of 4 weeks and are unaffected by maternal age.^[^[qv: 14]^]^ This nondestructive and highly accurate method is well suit for identifying single trophoblastic cells with high quality for molecular analysis.

Determining the fetal origin of putative cells is a prerequisite for using these cells in prenatal testing. We found that most of ER^high^ cells were β‐hCG and HLA‐G positive. Moreover, ER^high^ cells were also positive for Y chromosome in pregnancies with a male fetus in FISH analysis. All these findings suggest that most of ER^high^ cells were of fetal origin. In fact, it is not the phenotype, but the STR and SNP genotypes of putative cells that represent the gold standard for confirming their fetal origin. Currently, only one study has closely investigated the fetal origin of trophoblastic cells by sequencing a large panel of STRs and SNPs.^[^[qv: 15]^]^ The successful detection of STRs of rare trophoblastic cells in our study further supports the fact that ER‐LDA is valid in obtaining pure fetal cells and that it is able to obtain fetal genetic information precisely by analyzing these rare fetal cells. With a high purity of fetal cells, the accuracy of fetal genotyping is comparable to that obtained by invasive strategies and may be superior to cffDNA.^[^[qv: 28]^]^

Methods for prenatal diagnosis of many monogenic diseases with high incidence still rely on invasive procedures. Because of the limited amount and fragmented nature of fetal cfDNA, the cffDNA‐based test has several limitations for detecting monogenic disorders.^[^[qv: 29]^]^ Few attempts have been made to investigate the role of cell‐based test in the detection of monogenic disorders.^[^[qv: 30,31]^]^ Cell‐based testing has several advantages over cffDNA‐based test, in that fetal cells provide pure fetal DNA of high integrity.^[^[qv: 32]^]^ A total of 11 pregnancies whose fetuses may carry thalassemia mutations were investigated: five were at risk for –^SEA^/ thalassemia and six were at risk for β‐thalassemia. Thalassemia‐related mutations could be reliably detected by analyzing these rare fetal cells. This is the first study to diagnose fetal thalassemia by using rare trophoblastic cells isolated from Pap samples. More work is ongoing to explore the possibility of utilizing this cell‐based test for diagnosing other genetic diseases.

Some important limitations have to be considered in our study. Before our screening and diagnostic platform can be used in the clinic, further studies with a larger sample size are required and should include more patients with diverse genetic abnormalities. Owing to the limited length of the amplified DNA, this assay is presently not applicable for long fragment Gap‐PCR for the detection of −α^3.7^/ and −α^4.2^/ thalassemia. Thus, a more powerful whole genome amplification method may be warranted to obtain a better quality of fetal DNA with an entire genome that is adequate for more molecular analyses.

Overall, the ER‐LDA single cell platform presented here offers a noninvasive method for acquiring valuable molecular information from fetuses who are at risk for monogenic disorders. ER‐LDA has high accuracy in identifying rare trophoblastic cells with high quality. This test based on rare cells has high diagnostic accuracy without safety risk for the fetus when compared with invasive procedures, giving it the potential to be used in the clinic. The findings of our study imply that detection of rare fetal cells in Pap samples by ER‐LDA holds great promise for identifying fetuses with thalassemia, and other congenital defects.

## 4 Experimental Section

##### Participants and Sampling

From October 2017 through May 2019, a total of 232 Pap samples, including 167 from singleton pregnancies and 65 from nonpregnant controls, were collected from Nanfang Hospital, Southern Medical University (Guangzhou, China). Clinical information, including maternal age, gestational week and genetic testing results, was also collected. All samples were confirmed to be free of sperm contamination under a microscope. Samples with sperm contamination or with damaged cells were excluded from the study. This study was approved by the Ethics Committee of Nanfang Hospital, Southern Medical University and prior informed consent was provided by all participants. Specimens were safely obtained using a cotton swab, as described previously.^[^[qv: 30]^]^ Briefly, the cotton swab was rotated 360° at the external os, as the routine of Pap sampling, and stored in 5 mL of liquid‐based cytology test (LCT) fixative solution (Hologic, Inc., Marlborough, MA). Samples were stored at 4 °C, transported to the laboratory using freezer packs and processed within 5 h. Cells were harvested by centrifugation at 4000 rpm for 5 min and the cell pellet was washed with phosphate buffered saline (PBS) twice. Following this, the cell pellet was re‐suspended in 2 mL of PBS for ER staining and single‐cell isolation immediately. The samples used for PCR analysis were stored at −80 °C and processed within 2 months of collection.

##### Cell Lines

Three human trophoblastic cell lines (HTR8‐S/Vneo, JEG‐3 and JAR) were purchased from the American Type Culture Collection. HTR8‐S/Vneo and JAR cells were cultured in RPMI‐1640 medium (Gibco, Thermo Fisher Scientifc, Inc.) and JEG‐3 cells in Dulbecco's modified Eagle medium (DMEM, Gibco, Thermo Fisher Scientifc, Inc.) containing 10% fetal bovine serum (FBS, Gibco, Thermo Fisher Scientifc, Inc.) and 1% penicillin/streptomycin (Gibco, Thermo Fisher Scientifc, Inc.). Both cell lines were cultured in an atmosphere of 5% CO_2_ at 37 °C. Trophoblastic cells were collected for spiked‐in experiments and LDA analysis.

##### DNA Extraction and Quantitative PCR

To assess the presence and approximate number of trophoblastic cells in Pap samples, the copy number of the SRY (sex determining region of chromosome Y) gene, representing the number of fetal cells in samples from pregnancies with a male fetus, was calculated by ddPCR and qPCR (Figure S1, Supporting Information). DNA was extracted from the cell pellets of 172 Pap specimens (107 from pregnancies and 65 from nonpregnant women) using the commercial TIANamp Genomic DNA Kit (TIANGEN, Beijing, China). The Y chromosome copy number was determined by Droplet Digital PCR (ddPCR, Bio‐Rad QX200, Hercules, CA, USA) and Quantitative PCR (qPCR, Roche Cobas Z480, Basel, Switzerland). For ddPCR, the 20 µL ddPCR mixture consisted of 10 µL of 2 × ddPCR EvaGreen Supermix (Bio‐Rad, Hercules, CA, USA), 500 × 10^−9^ m of SRY primers (Table S3, Supporting Information), and 100 ng of DNA template. The mixtures were transferred to sample wells of a droplet generator cartridge (BioRad, Hercules, CA, USA); 70 µL of Droplet Generation Oil was loaded into the oil wells, and ≈20 000 water‐in‐oil droplets were generated in each outlet well. After that, the droplets were transferred to a 96‐well plate and amplified with the following conditions: 95 °C for 5 min, followed by 40 cycles of 95 °C for 30 s and annealing temperature (Table S3, Supporting Information) for 30 s and 1 cycle of 72 °C for 5 min. After PCR, the 96‐well plate was loaded on the QX200 Droplet Reader (Bio‐Rad, Hercules, CA, USA) and the data were analyzed using QuantaSoft analysis software (Bio‐Rad, Hercules, CA, USA). For the SRY qPCR, the 20 µL reaction mixture consisted of 1 × SYBR Green master mix (Takara Bio, Da Lian, China), 500 × 10^−9^ m of primers, and 100 ng of DNA template. The PCR conditions for the qPCR were the same as those for the ddPCR.

##### Screening of Candidate Cells

Aliquots of 2 mL cell suspension from Pap samples were first filtered by a 40 µm cell strainer (MACS Smart Strainer) to remove large epithelial cells. The cell pellet was washed with PBS and incubated with 1 × 10^−6^ m ER‐Tracker Red (Keygen Biotech, Jiang Su, China) for 30 min and then stained with 300 × 10^−9^ m DAPI (Invitrogen, MA, USA) for 15 min. Cells were centrifuged and washed with PBS, and 70 µL of cell suspension was applied to a slide and left for 3 min for the cells to settle. Each slide was scanned and imaged using an EVOS FL automated Cell Imaging System (Thermo Fisher Scientifc, Massachusetts, USA) in two fluorescent colors (ER‐Tracker: Red, DAPI: Blue) and the bright field. Cells were screened at low power (× 10) magnification and identified at high power (× 40) magnification. The ER‐Tracker fluorescence intensity and cell size of candidate cells were measured using Celleste Image Analysis Software.

##### Linear Discrimination Analysis (LDA)

LDA was used to discriminate trophoblastic cells from maternal cells. Using the ER‐Tracker fluorescence intensity and cell size signature of each cell as input, the LDA was implemented in SPSS version19.0 (IBM Inc., Chicago, IL, USA). Trophoblastic cells from cell lines, squamous epithelial cells and leukocytes from healthy female individuals were used as the training cohort and the test cohort. The LDA algorithm for discriminating trophoblastic cells from epithelial cells and leukocytes was determined. Subsequently, this LDA algorithm was applied to the clinical sample cohort. The probability of each candidate cell to be predicted as a trophoblast cell was obtained.

##### Single Cell Picking, Immunofluorescence and Fluorescence In Situ Hybridization

70 µL of cell suspension was layered on a slide and the target cell was identified by fluorescence microscope and LDA analysis. The micropipette was moved into the microscopic field of view and was in close proximity to the target cell. Subsequently, the individual cell was aspirated into the micropipette and the aspirated liquid containing the individual cell could be transferred to a new slide. Individual cell was easily released by dispensation. After air drying, the slides were fixed with 4% (w/v) paraformaldehyde at RT for 30 min and permeabilized by 0.2% TritonX‐100 for 30 min at RT. After three washing steps with PBS, the cells were blocked with 2% BSA at RT for 30 min. The slides were incubated with 1 µg mL^−1^ of β‐hCG (Thermo Scientific, MA, USA) antibody overnight at 4 °C. The cells were washed three times with PBS for 5 min followed by incubation with 1:400 diluted secondary antibody (goat anti‐mouse FITC, Bioss, Beijing, China) for 2 h and DAPI for 15 min at RT in the dark. Images were captured using an Olympus BX51 (Tokyo, Japan) fluorescence microscope.

Furthermore, putative cells were analyzed by FISH using the VividFISH FISH CEP kit (GeneCopoeia, MD, USA) for the detection of chromosomes X and Y, according to the kit protocol provided. Briefly, slides were dried at 56 °C in an oven for 2 h and incubated with 2 × SSC (saline sodium citrate) at 37 °C for 30 min. The slides were then dehydrated by a graded ethanol series (70%, 85%, and 100%) for 3 min each at RT and dried in air. Subsequently, the slides were hybridized with FISH probe sets (FISH probe for chromosome X and Y mixture in hybridization buffer) at a denaturation temperature of 75 °C for 10 min and at a hybridization temperature of 42 °C for 18 h. After that, the slides were washed in 0.5 × SSC/0.1%NP40 at 48 °C for 5 min and in 2 × SSC at 37 °C for 10 min. Finally, the slides were counterstained with DAPI for 20 min and images were captured using an Olympus BX51 (Tokyo, Japan) fluorescence microscope.

##### Whole Genome Amplification

A total of 48 samples were processed and characterized immediately after collection from patients. ER^high^ cells in these samples were used for WGA. Candidate cells on the slides were individually picked using glass micropipettes (40 µm in diameter) and confirmed on a new slide. Single isolated cells were then transferred into PCR tubes containing 4 µL of PBS solution. These cells were amplified using a REPLI‐g Single Cell Kit (Qiagen, Hilden, Germany) according to the instructions. Briefly, PCR tubes containing single cells were centrifuged for 2 min, and incubated with 3 µL of denaturation buffer at 65 °C for 10 min. After that, 3 µL of stop solution and 40 µL of master mix were added, respectively. Subsequently, the tubes were incubated at 30 °C for 8 h for amplification.

##### Quality Assessment of WGA Products

First, the size distribution of the WGA products was evaluated using the Agilent 2100 bioanalyzer system (Agilent Technologies, Waldbronn, Germany). The WGA products were loaded on DNA 1000 Lab Chips according to the manufacturer's instructions. Briefly, 9 µL of gel‐dye mixture was loaded on the micro‐channels using a 1 mL syringe; 6 µL of ladder mixture or WGA samples were loaded on the ladder well and sample wells, respectively. Chips were mixed using a Vortex Mixer at 2400 g for 1 min and immediately processed by the bioanalyzer. The results were analyzed using Agilent 2100 Expert Software (Agilent Technologies, Waldbronn, Germany).

##### STR Analysis

For STR genotyping, WGA products from rare trophoblastic cells and maternal gDNA were investigated using a PowerPlex System according to the manufacturer's protocol, and 19 human STR loci were detected: D1S1656, D2S1338, D3S1358, D5S818, D6S1043, D7S820, D8S1179, D12S391, D13S317, D16S539, D18S51, D19S433, D21S11, CSF1PO, Penta D, FGA, TH01, vWA, and Penta E. The PCR fragments were analyzed using an ABI3130xl Genetic Analyzer (Applied Biosystems Inc., Massachusetts, USA) and alleles were identified using GeneMapper 4.2 (Applied Biosystems Inc., Thermo Fisher Scientific, Massachusetts, USA).

##### Fluorescence Activated Cell Sorting

For fluorescence activated cell sorting (FACS), cells from Pap samples were incubated with 2 µg mL^−1^ of FITC‐labeled anti‐HLA‐G (MEM‐G/11, Invitrogen, MA, USA) at room temperature (RT) for 3 h in the dark. Cells were washed with PBS twice and processed on a MoFlo XDP Cell Sorter (Beckman Coulter Inc., CA, USA). The data were analyzed using Summit 5.2 software and the separated HLA‐G positive cells were used for whole genome amplification.

##### Sequencing of Fetal Cells from Thalassemia Samples

Trophoblastic cells that were ER^high^/DAPI^+^ were retrieved and amplified, as described above, from pregnancies with a fetal risk of thalassemia. The WGA product was then purified using a MinElute Gel Extraction Kit (Qiagen, Hilden, Germany), and its concentration was determined by Qubit 3.0 (Invitrogen, Massachusetts, USA). Amplification of target regions was conducted using the primers and the PCR conditions listed in Table S3 (Supporting Information). The purified PCR product was analyzed with Sanger sequencing (Applied Biosystems Inc., Massachusetts, USA).

##### Whole Exome Sequencing

For each sample, 1 µg of WGA product was randomly fragmented by Covaris (Woburn, MA, USA). Fragmented DNA with an average size of 200–400 bp was selected using an Agencourt AMPure XP‐Medium kit (BeckmanCoulter, NSW, Australia). After the processes of end‐repair, 3′ adenylated, adapter‐ligation and PCR amplifying for the selected fragments, the PCR products were purified using an AxyPrep Mag PCR Clean Up Kit (Axygen, CA, USA). The purified products were used for hybridization with BGI hybridization kits. After that, the hybridization products were recovered using the AxyPrep Mag PCR Clean Up Kit. The PCR products were heat denatured and single‐stranded DNA was circularized by the splint oligo sequence. The single strand circle DNA (ssCir DNA) was used as the final library and qualified by QC. The circular DNA was then amplified with Phi29 DNA polymerase. The formed DNA nanoballs (DNB) were loaded on a patterned nanoarray. Finally, the pair‐end 100‐base reads were generated by sequencing combinatorial Probe‐Anchor Synthesis (cPAS) on BGISEQ‐500 platform (BGI‐shenzhen, china). The sequencing data were analyzed by bioinformatics tools and visualized in R, as described previously.^[^[qv: 10]^]^

##### Statistical Analysis

Continuous variables were presented as means ± SD, with *n* indicating the number of samples. Linear regression was used to analyze the associations between trophoblastic cell number and other variables. Mann‐Whitney U test was used in the genomic coverage analyses. *P* < 0.05 was considered statistically significant.

## Conflict of Interest

The authors declare no conflict of interest.

## Supporting information

Supporting Information
