# Supplementary material for: Nondestructive Identification of Rare Trophoblastic Cells by Endoplasmic Reticulum Staining for Noninvasive Prenatal Testing of Monogenic Diseases
Source: Adv Sci (Weinh). 2020 Feb 13;7(7):1903354. doi: 10.1002/advs.201903354 (PMC7141004; doi:10.1002/advs.201903354)
Supplement: Supplementary file 1 — Supporting Information [file ADVS-7-1903354-s001.pdf]

Copyright WILEY-VCH Verlag GmbH & Co. KGaA, 69469 Weinheim, Germany, 2020.

ADVANCED SCIENCE
Open Access

Supporting Information

for *Adv. Sci.,* DOI: 10.1002/advs.201903354

Nondestructive Identification of Rare Trophoblastic Cells by Endoplasmic Reticulum Staining for Noninvasive Prenatal Testing of Monogenic Diseases

*Yifang Huang, Bo Situ, Liping Huang, Yingsi Cao, Hong Sui, Xinyi Ye, Xiujuan Jiang, Aifen Liang, Maliang Tao, Shihua Luo, Ye Zhang, Mei Zhong, and Lei Zheng**

Copyright WILEY-VCH Verlag GmbH & Co. KGaA, 69469 Weinheim, Germany, 2020.

Supporting Information

Nondestructive Identification of Rare Trophoblastic Cells by Endoplasmic Reticulum Staining for Noninvasive Prenatal Testing of Monogenic Diseases

*Yifang Huang†, Bo Situ†, Liping Huang†, Yingsi Cao, Hong Sui, Xinyi Ye, Xiujuan Jiang, Aifen Liang, Maliang Tao, Shihua Luo, Ye Zhang, Mei Zhong, Lei Zheng **

Dr. Y. Huang, Dr. B. Situ, X. Ye, X. Jiang, M. Tao, S. Luo, Y. Zhang, Prof. L. Zheng
Department of Laboratory Medicine, Nanfang Hospital, Southern Medical University, Guangzhou, 510515, China
E-mail: nfyyzhenglei@smu.edu.cn
Dr. Y. Huang, Dr. B. Situ, X. Ye, X. Jiang, M. Tao, S. Luo, Y. Zhang, Prof. L. Zheng
Guangdong Engineering and Technology Research Center for Rapid Diagnostic Biosensors,
Nanfang Hospital, Southern Medical University, Guangzhou 510515, China
Dr. L. Huang, Y. Cao, Prof. M. Zhong
Department of Obstetrics and Gynecology, Nanfang Hospital, Southern Medical University, Guangzhou 510515, PR China.
Dr. H. Sui, A. Liang
Department of Laboratory Medicine, Dongguan Kanghua Hospital, Dongguan 523080, PR China.
† These authors contributed equally to this work.

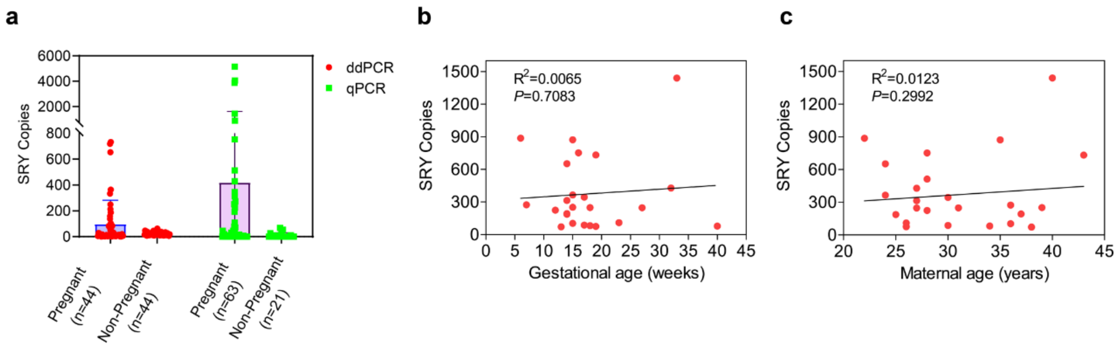

Figure S1. Y chromosome of fetal cells detected by ddPCR and qPCR. a) The number of fetal cells evaluated by ddPCR and qPCR via detection of the copy number of *SRY* in Pap samples from 107 pregnancies and 65 non-pregnant women. Ten of 44 samples from pregnant women in ddPCR (an average of 277 copies) and 20 of 63 in qPCR (an average of 1310 copies) show *SRY* positivity. The copy number of *SRY* is correlated to maternal age b) and gestational age c) using linear regression (the outliers are excluded). No significant correlation is observed.

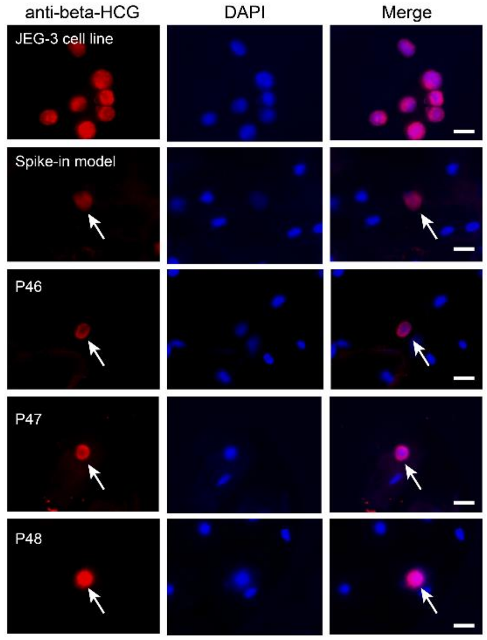

Figure S2. Immunostaining of trophoblastic cells in Pap samples. Representative

images of β-hCG staining for trophoblastic cells in three Pap samples are shown. The white arrow indicates the representative cells positive for β-hCG. Scale bar, 20μm.

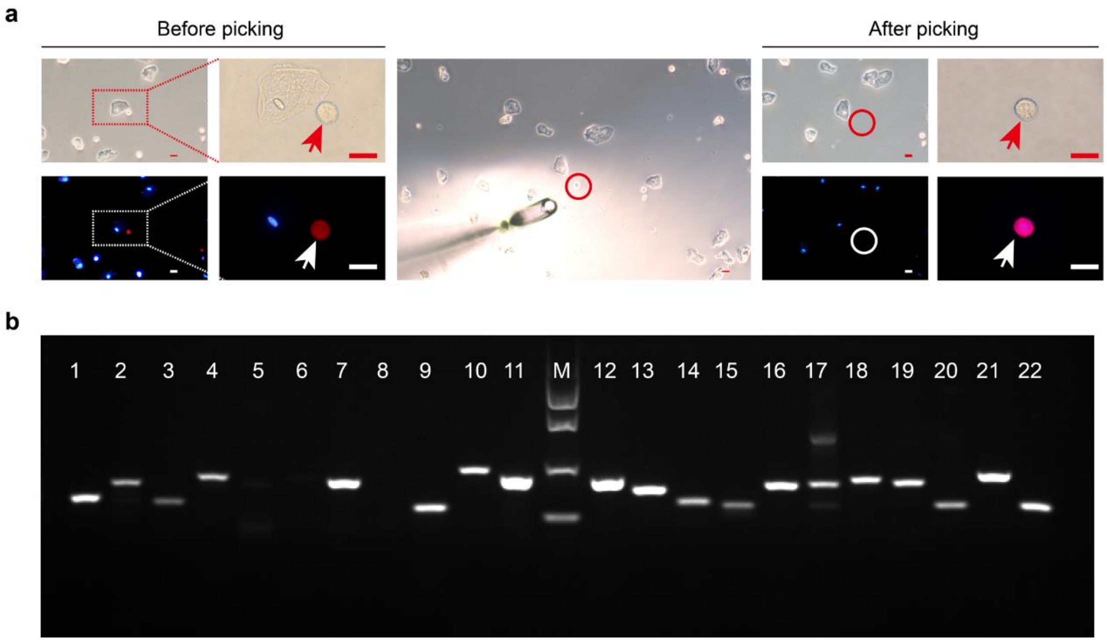

Figure S3. The process of single-cell isolation and the genomic coverage of WGA product. a) The process of single-cell isolation. Target cells are manual isolated by a micropipette. b) The genomic coverage of WGA product assessed by 22 genomic loci. 19 of 22 loci are detected in P26.

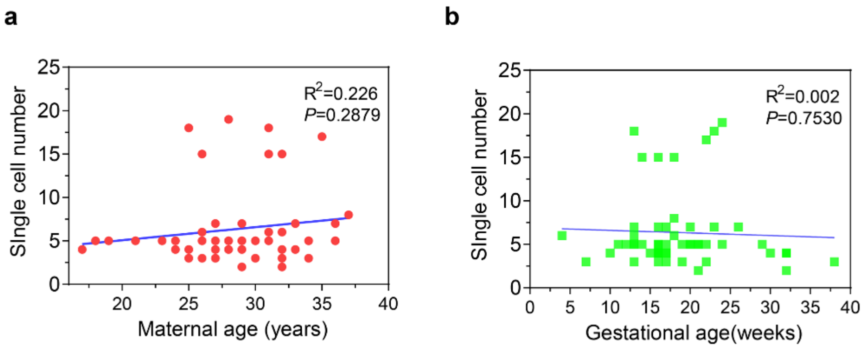

Figure S4. Correlating the number of rare trophoblastic cells and maternal age,

gestational weeks. Significant correlation is not observed in maternal age a) and gestational weeks b).

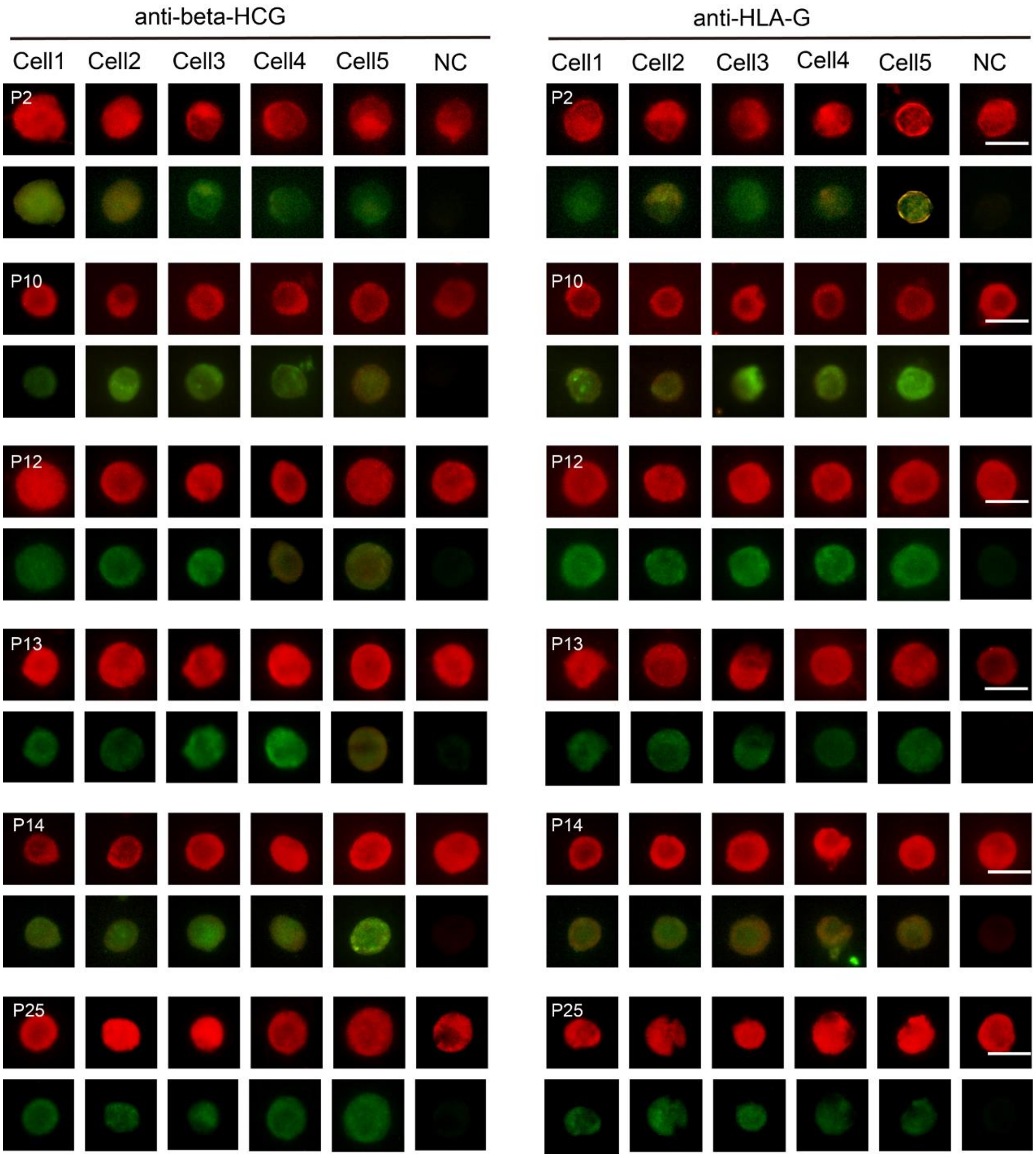

Figure S5. ER^{high} single cells analyzed by immunostaining. Twelve single trophoblastic cells are picked from each sample, five cells are stained with β-hCG (FITC, Green) and five stained with HLA-G (FITC, Green), two cells are used for

negative control (NC); ER-Tracker is shown in red. Scale bar, 20µm.

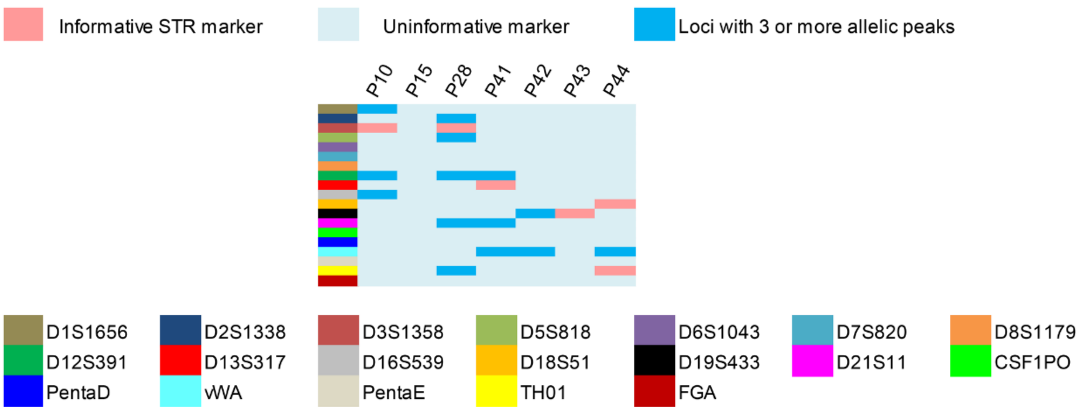

Figure S6. Cells with low LDA predictive probability and cells sorted by flow cell sorting assessed by STR. Cells with low LDA predictive probability in P10, P15, and P28 show maternal cell contamination in STR analysis. For flow cell sorting, 54, 69, 30 and 79 cells are separated in P41, P42, P43 and P44, respectively. These samples also show maternal cell contamination in STR analysis. The blue squares represent the loci with three or more allelic peaks.

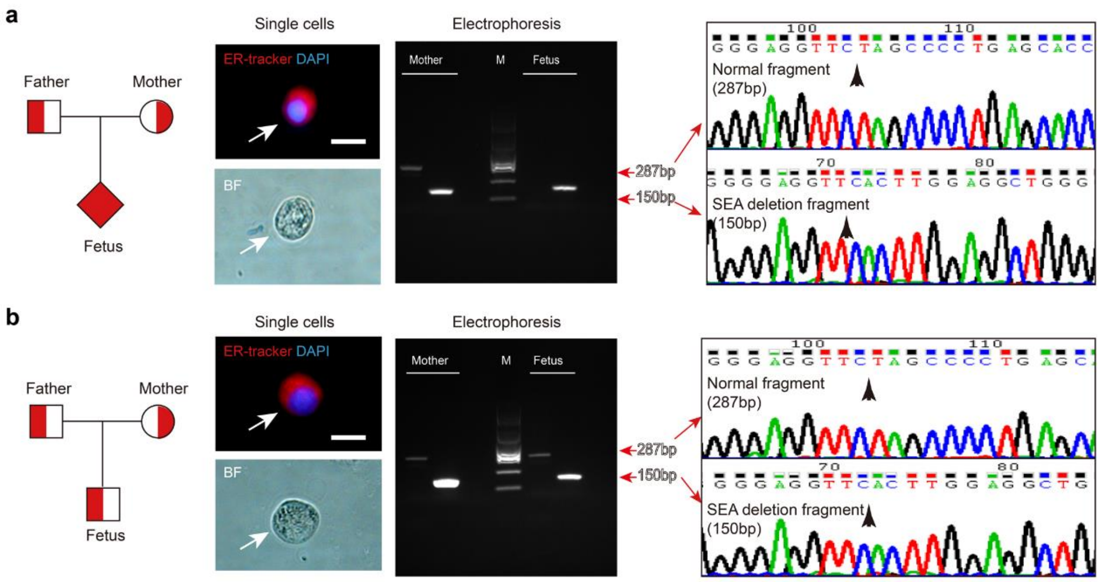

Figure S7. Rare fetal trophoblastic cells for diagnosis of SEA α-thalassemia. Another two cases (not shown in Figure 3) whose fetuses are at risk for $--^{SEA}/$ thalassemia are shown. a) The white arrow indicates the single cells isolated from P5. Photograph of agarose gel shows the $--^{SEA}/$ deletion detected by Gap-PCR assay. The deletion breakpoint of $--^{SEA}/$ mutation can be found by Sanger sequencing. b) The white arrow indicates the single cells isolated from P1. Photograph shows the $--^{SEA}/$ deletion detected by Gap-PCR assay. The deletion breakpoint of $--^{SEA}/$ mutation can be found by Sanger sequencing. Scale bar: 20 μm. Fully symbols indicate affected members who carry homozygous mutation. Half-filled symbols indicate members who carry heterozygous mutation. Squares indicate males, circles indicate females, and diamonds indicate individuals of unknown gender.

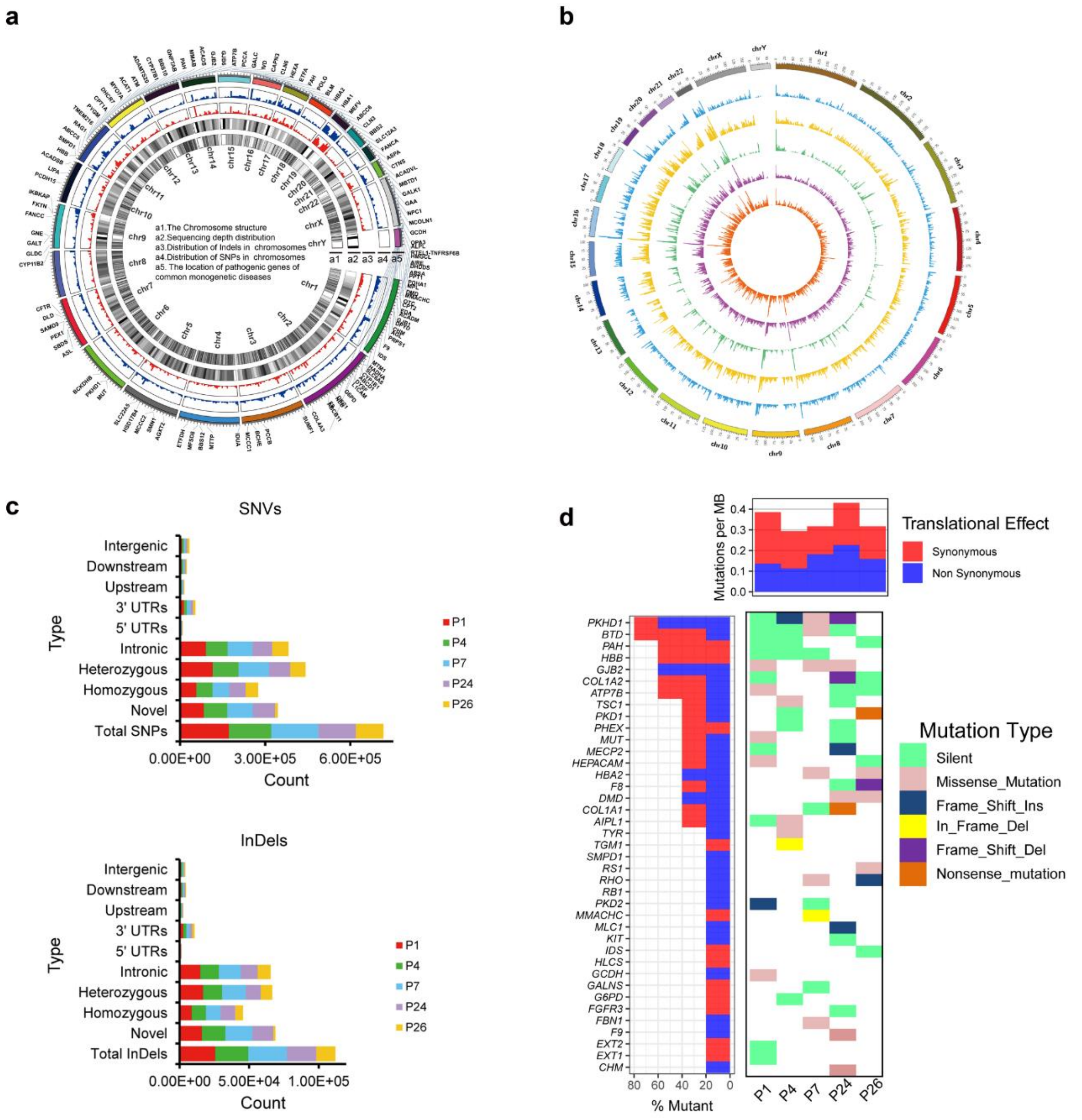

Figure S8. Whole exome sequencing for WGA products amplified from rare trophoblastic cells. a) Information on the whole exome sequencing for patient P1. The inner to outer circles represent the chromosomal structure, sequence depth, InDel distribution, SNP distribution and pathogenic genes of common monogenic diseases. b) Copy number profiles of five samples measured by WES. The inner to outer circles represent P7, P4, P26, P24 and P1. c) The total number and classification of variations. d) The variant profile for the 39 most common monogenic disease-associated genes.

The pathogenicity of these variants needs to be investigated further.

Table S1. Clinicopathological characteristics of 60 pregnant subjects.

Subjects	Age (years)	Gestational age(weeks)	Gravidity	Parity	Single cells	Diagnosis	Analysis by
P1	29	22^{+1}	3	0	5	Thalassemia carrier	WGA
P2	25	23^{+6}	2	0	18	Thalassemia carrier	WGA, Immunofluorescence
P3	32	32^{+5}	2	1	2	Normal pregnancy	WGA
P4	31	4^{+4}	0	0	6	Normal pregnancy	WGA
P5	30	29^{+2}	2	0	5	Thalassemia carrier	WGA
P6	30	16^{+5}	3	1	3	Thalassemia carrier	WGA
P7	28	16^{+1}	1	0	4	Normal pregnancy	WGA
P8	36	17^{+1}	3	1	7	Normal pregnancy	WGA
P9	29	26^{+3}	4	0	7	Normal pregnancy	FISH,WGA
P10	35	22^{+4}	2	1	17	Normal pregnancy	WGA Immunofluorescence
P11	30	19	2	0	5	Thalassemia carrier	WGA
P12	31	13^{+6}	2	1	18	Normal pregnancy	WGA, Immunofluorescence
P13	26	18	1	0	15	Normal pregnancy	WGA, Immunofluorescence
P14	31	16^{+2}	2	1	15	Thalassemia carrier	WGA, Immunofluorescence
P15	34	22^{+1}	6	3	3	Normal pregnancy	WGA
P16	25	19	1	0	3	Normal pregnancy	WGA
P17	32	16^{+1}	3	2	4	Normal pregnancy	WGA
P18	19	13^{+6}	1	0	5	Thalassemia carrier	WGA
P19	29	21^{+3}	2	1	2	Normal pregnancy	WGA
P20	37	18	5	1	8	Normal pregnancy	FISH,WGA
P21	18	21	1	0	5	Normal pregnancy	FISH,WGA
P22	27	7^{+2}	1	0	3	Normal pregnancy	WGA
P23	32	17^{+6}	4	1	3	Normal pregnancy	WGA
P24	33	15^{+2}	5	2	4	Normal pregnancy	WGA
P25	32	14^{+2}	2	1	15	Thalassemia carrier	WGA, Immunofluorescence
P26	28	24^{+1}	1	0	19	Normal pregnancy	WGA, Immunofluorescence
P27	32	18	5	2	6	Normal pregnancy	FISH,WGA

P28	26	13^{+2}	2	1	6	Normal pregnancy	WGA
P29	36	12^{+6}	3	0	5	Normal pregnancy	WGA
P30	25	32^{+6}	2	0	4	Thalassemia carrier	WGA
P31	27	13	1	0	7	Normal pregnancy	FISH,WGA
P32	26	38^{+2}	2	0	3	Normal pregnancy	WGA
P33	27	17	1	0	4	Normal pregnancy	WGA
P34	28	16^{+4}	2	1	5	Normal pregnancy	WGA
P35	29	21^{+2}	1	0	5	Normal pregnancy	FISH, WGA
P36	26	14^{+2}	2	0	5	Normal pregnancy	WGA
P37	24	20^{+5}	1	0	5	Normal pregnancy	FISH, WGA
P38	21	13^{+2}	2	0	5	Normal pregnancy	WGA
P39	31	16^{+2}	4	1	5	Normal pregnancy	WGA
P40	26	13^{+1}	1	0	3	Normal pregnancy	WGA
P41	30	16^{+4}	1	0	54	Normal pregnancy	Flow cytometry
P42	29	6^{+6}	2	1	69	Normal pregnancy	Flow cytometry
P43	36	$15^{+}4$	3	2	30	Normal pregnancy	Flow cytometry
P44	26	19^{+3}	NA [a)]	NA [a)]	79	Normal pregnancy	Flow cytometry
P45	29	36^{+2}	2	0	2	Normal pregnancy	WGA,
P46	23	10^{+2}	4	2	-	Normal pregnancy	β-hCG staining
P47	24	20^{+5}	1	0	-	Normal pregnancy	β-hCG staining
P48	26	18^{+6}	1	0	-	Normal pregnancy	β-hCG staining
P49	24	16	NA [a)]	NA [a)]	4	Normal pregnancy	WGA
P50	17	10	1	0	4	Thalassemia carrier	WGA
P51	31	20^{+1}	2	1	5	Normal pregnancy	WGA
P52	23	11^{+1}	3	0	5	Normal pregnancy	WGA
P53	34	24^{+4}	1	0	5	Normal pregnancy	WGA
P54	27	17^{+5}	1	0	5	Thalassemia carrier	WGA
P55	33	23^{+3}	3	1	7	Normal pregnancy	WGA
P56	27	30	1	0	4	Normal pregnancy	WGA
P57	29	20^{+2}	1	0	7	Normal pregnancy	WGA
P58	27	17^{+2}	2	1	4	Thalassemia carrier	WGA
P59	29	32	3	1	4	Thalassemia carrier	WGA
P60	27	16	1	0	7	Normal pregnancy	WGA

[a)]NA, Not available

Table S2. STR results of candidate trophoblastic cells isolated from Pap samples.

Patient	Age	Gestational age （weeks）	Single cells	*STR*			
				Total loci	Covered loci	Informative loci	Mismatch loci
P1	29	22	5	19	19	9	0
P3	32	32	2	19	19	0	0
P4	31	4	6	19	17	10	1
P6	30	16	3	19	7	2	1
P7	28	16	4	19	16	10	0
P8	36	17	6	19	12	11	0
P9	29	26	4	19	12	9	0
P10	35	22	5	19	16	4	3
P11	30	19	5	19	10	4	1
P12	31	13	6	19	17	12	1
P15	34	22	3	19	18	0	0
P16	25	19	3	19	7	6	0
P17	32	16	4	19	10	7	0
P18	19	13	5	19	13	6	1
P19	29	21	2	19	5	2	0
P20	37	18	5	19	6	4	0
P21	18	21	3	19	10	6	0
P22	27	7	3	19	7	4	0
P23	32	17	3	19	11	2	0
P24	33	15	4	19	13	11	0
P26	28	24	7	19	16	8	1
P27	32	18	3	19	10	8	0
P28	26	13	6	19	16	7	5
P29	36	12	5	19	6	4	1
P33	27	17	5	19	4	3	0
P40	26	13	5	19	5	4	0
P49	24	16	4	19	4	4	0
P50	17	10	4	19	8	3	0
P51	31	20	5	19	5	1	0
P52	23	11	5	19	7	2	1
P53	34	24	5	19	6	3	0
P55	33	23	7	19	4	2	0

Table S3. Primers used in the detection of *SRY* and diagnosis of thalassemia

For	Primer Name		Sequence (5'-3')	Size	Annealing temperature(℃)
Detection of *SRY*	ACTB	F	TGGCACCACACCTTCTACAA	116bp	62
		R	CCACTCACCTGGGTCATCTT		
	SRY	F	CCACTTACCGCCCATCAAC	104bp	60
		R	AGGTCTTTGTAGCCAATGTTACCC		
Diagnosis of thalassemia	HBB1	F	AGAAGAGCCAAGGACAGGTACG	353bp	61
		R	CTCTGTCTCCACATGCCCAGTT		
	HBB2	F	TCTGATAGGCACTGACTCTCTCTG	309bp	60
		R	GGGGAAAGAAAACATCAAGCG		
	HBB3	F	CATGCCTCTTTGCACCATTCTAA	568bp	60
		R	TGACCTCCCACATTCCCTTTTTAG		
	SEA-normal	F	GTGTTCTCAGTATTGGAGGGAA	287bp	58
		R	GACACGCTTCCAATACGCTTA		
	SEA-deletion	F	GTGTTCTCAGTATTGGAGGGAA	194bp	58
		R	CTTACTGCAGCCTTGAACTCC		
